# Supplementary material for: Are carbon emissions trading and green financial instruments synergistic? -Comprehensive quantitative research based on content analysis
Source: PLoS One. 2024 Mar 7;19(3):e0298601. doi: 10.1371/journal.pone.0298601 (PMC10919601; doi:10.1371/journal.pone.0298601)
Supplement: S1 Data — (ZIP) [file pone.0298601.s001.zip › Data/地级市Z_F工作报告.zip!/╒π╜¡/╠¿╓▌╩╨/└┤╫╘╣1⁄2╓┌║┼í╛┬φ┐╦╩2╛▌═°í┐_2017.docx]

各位代表：

现在，我代表台州市人民政府，向大会报告政府工作，请予审议，并请市政协委员和其他列席人员提出意见。

一、2016年和过去六年工作回顾

2016年，是“十三五”开局之年，是奋斗和收获的一年。市政府认真贯彻落实省委、省政府和市委的决策部署，以“民营经济创新发展、综合交通等基础设施建设、补齐关键领域短板”三大历史任务为总抓手，拉高标杆、争先进位，统筹推进各项工作，主要经济指标好于预期、高于上年、快于省均，收获了一份成色高、分量重的成绩单。

一是拉高标杆促赶超。围绕跻身全省经济总量第二方阵总目标，齐心协力抓发展，速度和质量明显提升。生产总值达到3843亿元，增长7.7%，扭转了增速落后全省平均的局面。农业保持全省领先，增加值增长4.4%。工业回暖明显，增加值增长8.6%，增速居全省第二。固定资产投资增长13.9%，增速居全省第二。财政总收入达到584亿元，增长8%，一般公共预算收入达到343亿元，增长10.7%，城镇和农村常住居民人均可支配收入分别达到47162元和23164元，增长9%和9.1%，增速均居全省前列。金融机构不良贷款率仅1.32%，为全省最低之一。

二是聚精会神抓项目。建立重大项目联席会议制度，实行季度集中开工，实施“166”重大项目前期攻坚计划，全市386个重点建设项目完成投资840亿元，增长40%。打响“交通建设大会战”，杭绍台高铁、路泽太高架等项目以超常规速度完成前期、陆续开工，市域铁路S1线、杭绍台高速、台金高速东延二期等项目顺利开工。台州机场改扩建前期取得重大突破。“十纵十横”干线公路网基本成型。实施市县联动招商和驻点招商，招引重大项目120多个，北航通航产业园、新吉奥汽车、氢能小镇等重大项目落户。彩虹无人机、中车台州配套产业园、吉利发动机、中国联塑台州生产基地等开工建设。吉利路桥汽车等一批重大产业项目蓄势待发、即将投产。

三是多措并举推改革。供给侧结构性改革成效明显。出台“1+29”系列政策。坚定不移去产能，清理“僵尸企业”28家；调节供需去库存，全市商品房去库存周期降至7.5个月，处于合理区间；积极稳妥去杠杆，规上工业企业资产负债率降至55%；加大力度降成本，减轻企业负担90多亿元。深化行政审批制度改革，“五张清单一张网”更加完善，成为国家级行政审批服务标准化试点，“最多跑一次”改革领跑全省。组建了一批国资平台。全面启动民间投资创新综合改革，积极探索和运用PPP模式，杭绍台高铁成为全国首条民营资本控股的高速铁路。农村“三位一体”改革纵深推进，市县乡三级农合联组建工作走在全省前列。

四是只争朝夕补短板。加快补齐体制机制等关键领域短板。市区融合取得决定性进展，在公交、交警一体化率先突破基础上，普通高中一体化顺利实施，财政、土地、规划等管理体制改革迈出实质性步伐。实施市区四大城建攻坚破难行动，打通13条断头路，内环路实现无障碍施工，完成90块闲置土地整治，改造城中村2100万平方米。“三区两市”协同发展加快推进。深入实施创新驱动战略，积极创建国家级高新区，高标准建设台州科技城，新引进清华台州创新中心、台州北大科技园等一批高端创新平台，全市各类科创平台达30个。全年新增发明专利授权量1534件、高新技术企业83家，新产品产值率达33.5%。大力改善城乡环境，建成美丽公路1351公里、绿道149公里，和合公园建成开园，被确定为全国大气和水环境质量改善明显的四个城市之一，生态环境状况指数居全省第二。

五是不遗余力惠民生。十方面民生实事圆满完成，民生投入继续增加。出台创业就业新政，推动大众创业、万众创新，新增市场主体8.9万户，增长44.8%，平均每天增加240多户。大力发展教育、卫生、文化、体育等民生事业，北师大台州附中建成开学，市妇女儿童医院如期结顶。低收入农户人均可支配收入增速居全省第二。出色完成G20杭州峰会维稳安保任务，社会和谐稳定。

2016年作为本届政府任期最后一年，我们全面完成了市人民代表大会确定的各项目标任务，实现了圆满收官。

各位代表！

回首过去六年路，充满曲折和坎坷。面对国内外经济下行的巨大压力，面对新旧动能转换的巨大调整，面对各种社会矛盾交织的巨大挑战，我们积极适应、把握和引领新常态，坚定不移打好转型升级组合拳，经济社会发展取得新成就。六年来，我们主要做了以下工作：

（一）千方百计扩投资稳增长。顶住经济下行压力，坚持稳字当头，出台工业、外贸、房地产等多项政策，建立“五帮一化”机制帮扶企业，积极扩大有效投资，经济保持稳健发展。生产总值年均增长7.5%；财政总收入和一般公共预算收入年均分别增长11.1%和13%；社会消费品零售总额年均增长13.1%；城镇和农村常住居民人均可支配收入年均分别增长9.9%和11.1%。固定资产投资累计9793亿元，年均增长19.4%。公路建设投资603亿元，是“十一五”时期的2.5倍。

台金高速东延段、沿海大通道等一批重大交通项目建成。金清新闸排涝二期完工，栅岭汪和洪家场浦排涝调蓄工程、东官河综合整治工程等加快推进，朱溪水库、市引水工程开工建设。三门核电一期即将投产，台州第二电厂、仙居抽水蓄能电站、中石化台州油库竣工投运。引进项目1011个，资金达1113亿元。直接融资1170亿元。设立11个市级产业基金，规模达84.3亿元。

（二）坚持不懈调结构促转型。优化产业结构，三次产业比例从6.6:51.4:42.0调整为6.6:42.8:50.6。建成粮食生产功能区66.5万亩、省级现代农业综合区10个。农产品浙江农博会金奖数保持全省第一。建成股份合作农场55家，培育国家级农民专业合作社示范社43家、省级131家。市粮食批发市场、台州中心粮库建成投用。基本完成农村宅基地确权登记发证，实现市县乡三级农村产权交易平台全覆盖。完成六大渔港经济区规划编制，建成标准渔港13座，“一打三整治”成效明显。出台“中国制造2025”台州行动计划，扎实推进“四换三名”。台州医药产业入选第六批国家新型工业化产业示范基地，温岭泵业等6个产业集群列入省现代产业集群提升示范区试点，头门港医化园区成为国家首批循环化改造示范试点，金属资源再生产业基地成为国家“城市矿产”示范基地。创成全国质量强市示范城市，成为国家级智能马桶产业质量提升示范区，“浙江制造”试点县数量全省第一。创建智能模具小镇等5个省级特色小镇。建成各类小微企业创业园95家。“个转企”近2万家。成为全国首批商标注册登记受理处，新增驰名商标37件、地理标志证明商标8件。新增上市企业18家，累计44家，新增新三板挂牌企业54家。发明专利授权量年均增长27.8%。全面深化对德合作，成为中德工业城市联盟“11+7”首批成员城市，浙江中德（台州）产业合作园挂牌。建筑业产值突破2000亿元，成为建筑强市。实施服务业强县（市、区）培育工程，10个省级现代服务业集聚示范区加快建设。万达广场、银泰城、新日用品商城等一批现代商贸设施建成投用。方林二手车市场被认定为省五星级文明规范市场，实现全市零的突破。天台山和神仙居景区创成国家5A级旅游景区，临海台州府城文化旅游区通过国家5A级景区资源质量评估。旅游总收入年均增长23%。成为国家电子商务示范城市、省级跨境电子商务试点市，浙江中非国际经贸港运营。培育淘宝村107个，数量居全国地级市首位。

（三）突出重点抓改革激活力。被国务院确定为小微企业金融服务改革创新试验区，列入国家部委的改革试点34项、省级改革试点62项，七大领域200项改革全面推进。小微金改形成全国可复制可推广的经验，率先在全国建设信用信息共享平台、设立信保基金，创建浙江（台州）小微金融研究院，发布全国首个小微金融指数，成为全国首个商标专用权质押登记受理点，办理量占全国的31%。国家民间投资创新综合改革和新型城镇化、循环经济、社会信用体系建设示范城市等国家级改革试点扎实推进。行政审批“1+7”系列改革和“五张清单一张网”建设全省领先，商事制度改革全国领先。市县同权改革不断深化，市本级审批事项减少60%，非行政许可审批事项全面取消。天台成立全省首个县级行政审批局。

（四）统筹城乡治环境提品质。编制台州城市群规划，完成城市总体规划修改。城市化水平由55.5%提高到61.3%，市区建成区面积由116平方公里扩大到145平方公里。中央商务区基本成型，商贸核心区、月湖绿岛新区、绿心生态区等加快建设。内环路建设接近尾声，台州大道、市府大道东延等一批骨干道路即将竣工。开通每日2趟台州至上海始发高铁列车。市规划展览馆、博物馆和客运总站等建成投用，市科技馆建成。省级海绵城市试点顺利推进，地下综合管廊一期工程开工建设。“多城同创”捷报频传，成功创建国家卫生城市、国家环保模范城市、国家森林城市、国家绿化模范城市和国家文明城市提名城市。铁腕推进医化行业整治提升，全市医化企业由1000多家整治到114家，退出恶臭项目216个，主城区告别化工恶臭。“五水共治”获省首批“大禹鼎”，国家“水十条”考核断面全部达标，农村生活污水治理实现行政村基本覆盖。“三改一拆”拆改总量和拆后土地利用率居全省前列，累计拆违1.2亿平方米、“三改”1.2亿平方米。“交通治堵”成效明显，市区公交分担率由不足5.5%提高到15.7%。深入开展美丽乡村建设，5个县（市、区）建成省级美丽乡村先进县，打造93个省级美丽宜居示范村，培育建成140个市级美丽乡村精品村，建成69个农家乐特色村。启动135个省级历史文化村落保护利用，古村落普查建档走在全国前列。6个中心镇成为省级小城市培育试点。培训农民31万人次。成为新农村电气化市。

（五）主攻沿海拓平台促开放。大力发展海洋经济，列为全省湾区经济发展试验区，编制完成台州湾、三门湾、乐清湾区域统筹发展规划，“三湾联动”格局加速形成。高水平规划、高起点建设562平方公里的台州湾循环经济产业集聚区，建成30平方公里核心区块，一个产城融合、高品质的产业集聚和对外合作大平台已经形成。汽车制造、高端装备、医药健康等产业发展成效明显，航空、新材料、新能源等新兴产业加快培育。玉环漩门三期、临海北洋涂一期、温岭担屿涂等项目完工，沿海围涂22.8万亩。加快建设136平方公里的头门港新区、36.9平方公里的温岭东部新区、32平方公里的三门滨海新城。头门港建成开港，台州人民期盼已久的金台铁路和沿海高速全面开工建设，从而拉开“一体两翼”市域大框架，集疏运体系日益完善。新增省级经济技术开发区3个。长江经济带区域通关一体化启动。浙台（玉环）经贸合作区加快建设，大麦屿港区列为国家一类开放口岸，对台直航常态化，玉环成为首批国家级海洋生态文明建设示范区。健跳港区实现临时对外开放。进出口行业诚信体系建设经验全国推广。大陈岛列为全省首个海洋开发与保护示范岛。

（六）注重民生办实事谋福利。全市财政民生投入1465亿元，年均增长16.9%。累计完成为民办实事项目141项，投入200多亿元。新增就业52万人，基本消除城镇“零就业家庭”。全面消除家庭人均年收入4600元以下贫困现象。积极推进教育现代化市创建，实现义务教育发展基本均衡县全覆盖，新增32所省级特色示范普通高中，全市学前教育毛入园率达98.8%，省标准化学校比例达94.2%，高考录取率达89.9%。台州学院应用型办学迈出扎实步伐，台州职业技术学院建成省示范性高职院校，台州广播电视大学搬迁新校园，台州技师学院获批筹建并开始招生。公立医院改革顺利推进，“双下沉、两提升”成效明显，分级诊疗和全科医生签约制度全面实施，千人床位数、医生数和护士数分别提高59%、42%和74%，人均预期寿命达79.12岁，主要健康指标居全省中上水平。组建市中心医院（台州学院附属医院），恩泽医院建成投用，市第二人民医院顺利迁建，市公共卫生中心（食品药品检验检测中心）等加快建设。完成农贸市场改造提升三年任务，创建78家省级放心农贸市场。稳妥实施单独两孩、全面两孩政策，出生人口性别比实现“六连降”。文化产业稳步发展。国家公共文化服务体系示范区创建扎实推进，建成933个农村文化礼堂。台州府城墙入选中国世界文化遗产预备名录，“三门祭冬”列入联合国非遗代表作名录。仙居被命名为“中国慈孝文化之乡”。实现省体育强县全覆盖，成功举办两届台州国际马拉松比赛，被中国田径协会评为银牌赛事，市少体校（全民健身中心）建成投用。建成城乡社区居家养老服务照料中心3544家，居全省第二。基本养老、医疗保险参保扩面，城乡养老和医疗保险并轨提标，新增基本养老保险参保67万人、基本医疗保险参保97.9万人。交付保障房2.3万套。市儿童福利院建成投用。开展第三次农业普查，基层统计基础增强。浙江佛学院天台宗佛学院、浙江道教学院建设通过评估，济公故里成为“海峡两岸交流基地”。长潭水库38米线以下移民动迁安置全面完成。“智慧水务”加快建设，市应急（防汛、森林消防）指挥中心和渔船安全救助信息系统基本建成，连续11年台风灾害影响人员零死亡。外事侨务、气象、地方志等工作取得新成绩。妇女儿童、残疾人、老龄、老干部、关心下一代、慈善等工作不断加强。

（七）创新治理强法治促和谐。自觉接受市人大依法监督、市政协民主监督和社会监督，办理市人大代表议案建议1619件、市政协委员提案2083件。认真落实中央八项规定精神，严肃查处发生在群众身边的不正之风，依法惩处一批腐败分子。重视行政监察和审计监督。全市“三公”经费下降63.6%。注重发挥法律顾问等专家团队决策咨询作用。基层协商民主有序推进。“六五”普法和公共法律服务体系建设扎实有效，《台州市城市市容和环境卫生管理条例》《台州市城乡规划条例》两部地方性法规议案提请市人大常委会审议通过。建立全市统一政务咨询投诉举报平台，12345市长热线服务质量居全国地级市第六。平安台州建设不断加强，实现“四连创”，获得“平安铜鼎”。社会治理创新扎实推进，立体化治安防控体系逐步建立，刑事案件、交通和消防事故数逐年下降，安全生产控制指标持续下降。社区矫正规范化建设经验全国推广。人民防空、反走私综合治理和海防工作走在全省前列。食品安全公众满意度逐年上升，县级食品安全检验检测资源整合工作全国推广。大力加强国防动员和后备力量建设，支持驻台部队建设，连续4次获得全国双拥模范城称号。援疆等对口帮扶和支援工作走在全省前列。

各位代表！

回顾这些年不平凡的经历，我们感慨万千。过去六年，是我们在全球性经济危机的深层次影响下，过坎爬坡、负重前行的六年；是我们在经济发展新常态下，痛定思痛、艰难转型的六年；是我们在人民群众日益增长的良好生态环境和社会公平正义需求下，壮士断腕、大治环境的六年；是我们在“不进则退、慢进也是退”的激烈区域竞争中，磨砺成长、重振信心的六年。我们经受住了经济下行带来的历史性考验，保持了经济稳健运行，为赶超发展奠定了坚实基础。经过努力，小微金融改革成为全国样板，综合信用指数全国地级市排名第三，纳税人满意度全国地级市排名第一，民生发展位列全国地级市第十三，创新指数居全国大中城市第三十一，法治政府建设居全国大中城市第十，城市空气质量跻身全国大中城市前十。成绩来之不易，经验弥足珍贵。

——面对经济下行巨大压力，我们必须保持定力、创新转型，坚定不移发展实体经济。以民营为主体的实体经济是台州最厚重的底牌和最靓丽的特色。这些年，不管金融危机多么风高浪大，我们始终坚持稳字当头，坚持工业强市不动摇，时刻牢记习近平总书记当年对台州的嘱托，不遗余力打造先进制造业基地，持之以恒打好“拆治归”转型升级系列组合拳，实现“凤凰涅槃”，发展保持中高速，质量迈向中高端。历经磨难，台州实体经济的脊梁仍然坚挺。

——面对经济下行巨大压力，我们必须锐意改革、奋发有为，坚定不移优化发展环境。经济发展进入新常态，营商环境对区域发展尤为重要。我们自觉将政府有为之手和市场无形之手有机结合起来，不断深化小微金融改革，努力优化金融环境；大力加强科创平台建设，努力优化创新驱动环境；以自我革命精神推动政府自身改革，努力优化政务生态环境；着力加强法治政府建设，努力优化法治环境。如果将台州经济比作汪洋大海中的一艘航船，那么金融就是压舱石，科技创新和政府自身改革就是双引擎，法治社会建设就是规则系统，四个方面协调发力，这艘航船就能劈波斩浪、行稳致远。

——面对经济下行巨大压力，我们必须着眼长远、集聚资源，坚定不移改善交通条件。台州要赢得区域发展主动，必须加快融入长三角都市圈。这些年，我们始终把交通建设放在突出位置，以功成不必在我的胸襟，“勒紧裤带、砸锅卖铁”的决心，全面打造升级版的互联互通，一批事关全局和长远的重大交通项目相继开工，“三区两市”协同、“三湾联动”和全市域统筹发展的条件更加充分。在不远的将来，台州内外交通条件将发生根本性改变。

——面对经济下行巨大压力，我们必须秉持“民之所需、为政所向”，坚定不移提升群众获得感。人民群众对美好生活的向往就是我们的奋斗目标。富裕起来的台州人民对良好生态环境和教育卫生条件的渴望从未像现在这么强烈。这些年，我们超常规加大教育、卫生等投入力度，民生短板加快补齐。我们义无反顾推进专项工作，以壮士断腕的决心治气治水，生态环境明显改善。空气质量走在全国前列，台州蓝成为常态，这成了老百姓最有获得感的福利。

——面对经济下行巨大压力，我们必须克难奋进、实干担当，坚定不移加强政府自身建设。干事担当的干部队伍是推动发展的根本保证。这些年，我们认真落实全面从严治党要求，切实加强勤政廉政建设。坚持以实干论英雄，始终不渝优作风、提效能，坚持不懈重实干、勇担当。在赶超发展的新长征路上，台州铁军正充满自信、意气风发地阔步前行。

各位代表！

过去六年我们所取得的成绩和进步，是省委、省政府和市委坚强领导的结果，是市人大及其常委会、市政协有效监督和大力支持的结果，是全市人民励精图治、奋力拼搏的结果。在此，我代表市人民政府，向全市人民和外来建设者，向人大代表、政协委员，各民主党派、工商联、人民团体、社会各界人士和离退休干部，向驻台人民解放军和武警部队官兵、在台省部属各单位，向关心、支持台州发展的海内外朋友们，表示衷心的感谢！

在看到成绩的同时，我们也清醒认识到发展中还有不少问题，政府工作也存在不足。一是对照先进和标杆，我们不仅存在经济总量差距，还存在人均指标差距，不仅有发展速度差距，还有质量效益差距。二是科技和人才短板突出，高端开放合作大平台比较缺乏，民间投资增速放缓，新旧动能接续转换还需付出更大努力。三是中心城市辐射带动能力不强，制约发展的体制机制障碍有待进一步消除，城乡统筹发展仍需加强。四是教育、卫生、文化等基本公共服务供给不足，保障和改善民生的任务还很繁重。五是安全生产事故多发势头没有得到根本遏制，基层社会治理能力和水平有待提高。六是干部能力、作风还有不适应问题，一些地方懒政怠政依然存在，消极腐败现象时有发生。我们一定要直面问题，克难攻坚，全力以赴做好政府工作，不辱历史使命，不负人民重托。

二、今后五年发展目标

雄关漫道真如铁，而今迈步从头越。站在新的历史起点上，我们无比欣喜地感到，台州发展的又一个春天已经来到。我们正挺立在过坎爬坡、喜迎曙光的台阶上，正拼搏在腾笼换鸟、转型升级新的层面上，正奋进在赶超发展、再创辉煌的跑道上。台州潜力无限、前景美好！有党中央、国务院的坚强领导，有省委、省政府和市委的正确部署，有历年来的坚实积累，有善于创造的台州人民，我们完全有信心有能力实现赶超发展。我们要进一步增强台州自信，强化抢抓机遇意识、赶超进位意识、裂变扩张意识、大抓项目意识、主动担当意识，干出台州力度，跑出台州速度，奋力跻身全省经济总量第二方阵。

今后五年台州发展的指导思想：高举中国特色社会主义伟大旗帜，以马克思列宁主义、毛泽东思想、邓小平理论、“三个代表”重要思想、科学发展观为指导，深入贯彻习近平总书记系列重要讲话精神，以“五位一体”总体布局和“四个全面”战略布局为统领，以创新、协调、绿色、开放、共享五大发展理念为引领，以“八八战略”为总纲，坚持以人民为中心的发展思想，在新的历史方位下，拉高标杆、争先进位，勇立潮头、裂变发展，奋力完成三大历史任务，高水平全面建成小康社会，为中国道路、中国方案的浙江鲜活样板提供台州模块、台州经验，努力把台州建设成为独具魅力的“山海水城、和合圣地、制造之都”。

“山海水城、和合圣地、制造之都”，既是发展定位，也是战略目标。建设独具特色的“山海水城”，就是要立足于台州独特的自然禀赋，以生态宜居为特色，打造山海相依、城水相宜、人水相亲的城市品质。建设底蕴深厚的“和合圣地”，就是要立足于台州深厚的历史积淀，以和合文化为精髓，打造古今交融、和合共生、包容大气的城市人文。建设实力强劲的“制造之都”，就是要立足于台州扎实的产业基础，以先进制造为方向，打造高新高端、智能智慧、集聚集群的产业高地。

根据上述指导思想和战略目标，我们要围绕三大历史任务，深化供给侧结构性改革，打好转型升级组合拳，在全面完成“十三五”规划基础上，努力实现“一个跨越”“两个突破”“三个倍增”“四个明显提高”，即到2021年，全市生产总值跨越5000亿元大关，达到5500亿元，年均增长7.5%；工业增加值突破2000亿元，年均增长8%以上，一般公共预算收入突破500亿元，年均增长7.5%以上；固定资产投资、研发经费支出和发明专利授权量、旅游总收入等指标倍增，实现“翻一番”；城市化水平、民生投入、城镇和农村居民收入明显提高，城市化率达到66%以上，新增财力三分之二以上投向民生领域，城镇和农村常住居民人均可支配收入分别达到69000元和34800元。

一是奋力完成民营经济创新发展的历史任务，培育赶超发展新动能。以振兴实体经济为根本，壮大科技支撑，优化产权结构，创新体制机制，营造“亲”“清”新型政商关系，激发和保护企业家精神，推动民营企业薪火相传。深入实施“中国制造2025”台州行动计划，突出先进制造，大力弘扬工匠精神，打响“台州制造”品牌。培育一批拥有国内市场话语权、比肩国际先进水平的大企业大集团，形成10家以上百亿工业企业、五大千亿产业集群，打造国际“汽车城”“航空小镇”和“中国绿色药都”。

二是奋力完成综合交通等基础设施建设的历史任务，构建赶超发展新支撑。系统推进交通、城建、水利、通信等重大基础设施建设。大手笔构建海陆空多元立体、便捷通畅的交通体系。推动以头门港为龙头的台州港“一港六区”综合开发。杭绍台高铁、金台铁路、沿海高速、杭绍台高速、市域铁路、路泽太高架、现代有轨电车等建成通车，加快形成15分钟市区交通圈、30分钟“三市”同城交通圈、1小时市域交通圈、连接省内和长三角主要城市1至3小时交通圈。完成台州机场改扩建，建设一批通用机场。以中心城市为龙头，有序推进城市基础设施建设，高水平推进城乡建设。推进“五湖四环、一廊多带”都市区国家级水利风景区建设，全市域构建水上台州，积极创建全国水生态文明城市。大力推进信息化基础网络建设，打造全国一流、全省领先的“智慧台州”。

三是奋力完成补齐关键领域短板的历史任务，增强赶超发展新优势。以更大的决心和力度精准补齐短板，着力解决城乡区域发展的不平衡问题、平均数背后的大多数问题、指标外的群众真切感受问题，决不把脏乱差、污泥浊水、违法建筑带入全面小康。推进体制机制创新，理顺管理体制，加快市域中心城市人口集中、人才集聚，提高首位度和辐射力。坚持普惠性、保基本、均等化、可持续方向，发展人民满意的教育，建成教育现代化市；实施“健康台州2030”行动规划，创建国家健康城市示范市和国家食品安全城市；大力发展文体事业，创成国家公共文化服务体系示范区和省体育强市，努力实现基本公共服务全覆盖；加强社会治理创新，提升平安创建水平，切实增强人民群众安全感和幸福感。

三、2017年重点工作

今年是党的十九大召开之年，是供给侧结构性改革的深化之年，是台州走好赶超发展新长征路的起步之年，做好各项工作意义重大。主要预期目标为：生产总值增长7.5%以上，一般公共预算收入增长7.5%以上，固定资产投资增长12%以上，外贸出口适度增长，货物出口占全国比重8.6‰以上，社会消费品零售总额增长10%以上，城镇和农村常住居民人均可支配收入分别增长7.5%以上和8%以上，单位生产总值能耗下降和主要污染物减排完成省定任务。为实现上述目标，我们必须认真落实市五届一次党代会精神，坚持稳中求进、好中求快，以提高发展质量和效益为中心，以供给侧结构性改革为主线，以“大抓项目年”活动为载体，加快实现赶超发展。重点抓好以下六方面工作：

（一）聚焦实体经济，提升产业层次。

实体经济是台州赶超发展的坚实基础。要联动推进产业、企业和平台发展，促进覆盖一二三产业的实体经济蓬勃发展。

全面实施“改一强二兴三”。转型升级农业。完善强农惠农政策，推动农业供给侧结构性改革，建设品牌农业强市。认真落实粮食安全县（市、区）长责任制，确保全市38.83万吨地方储备粮规模、仓储、费用“三落实”。全面推进农业“一区一镇”建设，全力创建农产品质量安全放心市。加快农业“接二连三”，增加农业综合开发投入，发展观光农业、创意农业等新业态。扶持现代种业发展。培育新型农业经营主体，发展股份制家庭合作农场，基本完成农村土地承包经营权确权登记颁证，继续完善“三位一体”农民合作经济组织体系。推进创新农作制度示范乡镇建设。大力发展海洋经济，推动省渔业“两区一县”创建，发展绿色生态渔业，推进渔场修复振兴。做强先进制造。积极实施先进制造业“十大行动”。推动工业化、信息化深度融合，发展“互联网+”，提升汽车及配件、医化、模具、泵与电机、缝制设备、休闲用品等传统产业。大力发展高端装备、航空、新材料、新能源等战略性新兴产业，培育工业机器人、智能工厂、智慧管网等智造产业。开工建设北航通航产业园、氢能小镇、新能源产业园等项目。加快推进彩虹无人机、中车台州配套产业园和修造基地、新吉奥汽车、吉利汽车临海产业园扩建等重大项目。实现吉利V汽车下线、巨科铝轮毂量产、三门核电一期投运。促进军民产业融合，争创军民融合示范区。推进建筑工业化。壮大第三产业。大力发展工业设计、现代物流、软件和信息服务业等，加快生产性服务业向专业化和价值链高端延伸。加快大数据、云计算、物联网应用，发展新兴业态和分享经济。保持房地产业平稳健康发展。促进专业市场提档升级，大力发展会展经济。推动服务业模式创新和跨界融合，发展医养结合、健康养老、文化创意、体育休闲等产业。实施“放心消费在台州”行动，扩大数字家庭等信息消费，推动生活性服务业向精细化和高品质转变。积极顺应大众旅游时代到来，做好全域旅游顶层规划设计，梳理谋划一批凸显区域特色的大项目、好项目，创新旅游业态，促进产业融合，打响台州旅游品牌，全力争创国家全域旅游示范区，打造国内外知名旅游目的地。

大力支持企业做强做优。实施上市公司和骨干企业扩张裂变倍增行动，新增上市企业5家、新三板挂牌企业20家，鼓励上市企业开展兼并重组。加快培育“三名”企业，新增市级以上“三名”培育企业12家，总数达110家，着力形成一批行业龙头骨干企业。积极发展总部经济。滚动推进小微企业三年成长计划，完成“个转企”1200家以上、“小升规”150家以上。围绕“专、精、特、新”，培育中小企业“隐形冠军”和行业“单打冠军”。大力推动企业管理创新，深入实施“128”股改行动计划，出台配套政策，鼓励支持民营企业引入战略投资、优化股权和债权结构，强化法人治理，建立现代企业制度，新增股份制企业200家。大力支持企业开拓市场，鼓励企业以“一带一路”主要节点城市和港口为布局重点，加快“走出去”步伐，深化国际产能合作，创新服务贸易方式，促进外贸回稳向好。加大支持帮扶企业力度，进一步清费减负，降低经营成本。企业家是台州最为宝贵的战略资源，要高度重视企业家队伍建设，培养一批新生代企业家。

扎实推进平台建设。推动台州湾区经济发展试验区建设。以台州湾循环经济产业集聚区为核心，大力推动集聚区东部新区建设，拓展提升沿海开发平台。整合提升各类经济开发区、产业集群等平台。深化特色小镇建设，加快无人机国际航空小镇、临海国际医药小镇等高端制造小镇建设。按照集聚、集约发展要求，建设提升各类小微企业园100家。大力推进以对德合作为重点的全方位对外开放，加快中德（台州）产业园等对外合作园区建设，办好中德工业城市联盟大会。推进跨境电商产业园建设。谋划建设综保区和综合物流园区，头门港成为省级经济技术开发区。加快国际贸易“单一窗口”和通关一体化落地，推进公用型保税仓库等综合服务平台建设，引进培育若干外贸综合服务企业。加快推进口岸查验基础设施建设。积极申报国家级对台经贸合作区，加快建设海峡两岸（玉环）商品交易物流中心。

（二）聚焦创新转型，加快动能转换。

创新转型是台州赶超发展的根本路径。要深入实施创新驱动战略，推动新旧动能平稳接续、协同发力。

高度重视科技创新。实施科技创新“115”行动计划，加快建设“一城一区一廊”创新大平台，积极引进大院名校共建创新载体，充分发挥浙大台州研究院、台州学院等一批高端科创平台作用，深化与中国药科大学等战略合作，加速科技成果高效集聚和转化。加强知识产权保护。实施高新技术企业和科技型中小微企业“双倍增”计划，新认定国家级高新技术企业120家以上，新增省级科技型中小企业500家以上、省级以上技术中心5家。鼓励创办各类众创空间，推动大众创业、万众创新。加大高层次人才引进和培养，推进人才管理改革试验区建设。

深入推进“三强一制造”。建设质量强市，统筹产品、服务、环境、工程四大关键领域，深化全国质量强市示范城市建设。建设标准强市，编制台州特色标准体系规划，加快千亿级主导产业和百亿级新兴产业标准研制，争取智能马桶等专业标准化技术委员会落户台州。建设品牌强市，争创全国知名品牌创建示范区。落实“浙江制造”品牌建设三年行动计划，支持“智能马桶”“黄岩模具”等集群品牌发展，新增“浙江制造”认证企业6家，打响“好产品台州造”。

坚决打好“拆治归”组合拳。深化“五水共治”，坚决完成剿灭劣V类水任务，完善“河长制”，打通104条断头河，开展农村生活污水治理提标改造。强势推进“治危拆违”专项行动，深化“三改一拆”，消除违法建筑，整治城乡危旧房，启动230个城中村改造，着力推进“无违建”创建。加快拆后土地利用和闲置土地消化，盘活存量建设用地。建设“治土”先行区，开展土壤污染风险管控与治理修复。全面推进“五气共治”，打好蓝天保卫战。加大招商引资力度，把招商引资作为“一号工程”，优化投资环境，通过对接央企、利用外资、台州商人回归等途径，实现精准招商、产业链招商，推动产业、资本、人才、科技全方位多领域引进和回归。加强异地商会建设，充分发挥异地商会在招商引资中的重要作用。全年实现省外到资340亿元。

（三）聚焦中心城市，统筹市域发展。

中心城市是台州赶超发展的有力支撑。要发挥综合交通等重大基础设施项目带动作用，增强集聚能力，提升市域综合竞争力。

高水平建设中心城市。围绕建设“山海水城”目标，加快打造品质城市，让居民望得见山、看得见水、记得住乡愁。保持组团式城市发展形态，强化规划引领，着力提升城市规划水平，优化城市空间结构和布局，深入研究重点区块、重点项目城市设计，加快实现城区控规全覆盖、城市设计全贯穿。着力推进“多规合一”，编制台州水城、铁路中心站新区等规划，大力推进椒江“一江两岸”综合开发。以“810”美丽城市重点项目建设为抓手，加快中央商务区、商贸核心区、永宁江两岸、飞龙湖生态区、月湖绿岛新区等建设。大力推进城市有机更新，实施开发区东部区块、椒黄路老城区等更新改造。全面整治城市主要入城口，扮靓城市大门。统筹地上地下空间综合开发，推动人防工程多元利用，加快地下综合管廊和海绵城市建设。以综合行政执法改革为契机，推动城市精细化管理，实施城市“绿化、亮化、美化”提升工程。建设智慧城市，启动省级智慧交通示范项目建设。提升住宅小区物业管理水平。城市文化是构建城市特色的基因，要充分挖掘和合文化、章安古镇、东吴卫温船队首航台湾等特色内涵，融入城市建设，延续历史文脉，打造一批历史文化街区，留住城市记忆。倡导文明言行，培育文明新风，提升市民素养，确保通过国家卫生城市复审，创成全国文明城市。

加快综合交通等项目建设。顺应全省港口一体化进程，以头门港二期开工为契机，加快港口基础设施建设，完善集疏运体系，实现港产城融合发展。按照“三区融合”、“三区两市”协同发展和全市域统筹发展的要求，进一步拓展内通外联的交通网络。沿海高速完成形象进度73%以上，乐清湾段年底主体工程建成。内环路建成通车，台州火车站公交枢纽和新客运南站公交枢纽建成投用。杭绍台高铁全线动工。加快建设市域铁路S1线、金台铁路、杭绍台高速、台金高速东延二期和市区连接线、路泽太高架、现代大道、228国道温岭泽国至大麦屿疏港公路等项目。开工建设市域铁路S2线、现代有轨电车、绕城高速南线、杭温高铁、上三高速东延等项目。开工建设台州机场改扩建工程和集聚区通用机场。实施水利“五大百亿工程”，加快朱溪水库、方溪水库、东屏水库、市引水工程、栅岭汪和洪家场浦排涝调蓄工程、东官河综合整治、青龙浦排涝、温岭南排等重点项目建设。开工建设洪家场浦强排、黄岩北排、永宁江强排、灵江扩排挡潮、七条河拓浚等工程。建成甬台温天然气和成品油管道等项目。

建设“美丽台州”。坚持“三湾联动”和“三区两市”协同发展，加快临海、温岭两个副中心城市发展，努力构建城市功能互补、通勤时间合理、公共服务一体的协同发展格局。实现玉环撤县设市，支持天台、仙居、三门特色发展。推进小城市培育和中心镇建设。加快大陈岛“三区三基地”建设，支持市区西部山区发展。深化小城镇环境综合整治，创建美丽集镇。加强历史文化村落保护，实施浙派民居改造，启动建设17个省级美丽宜居示范村，培育建成10个市级美丽乡村示范乡镇，让美丽乡村与美丽集镇、美丽城市交相辉映。全面推进“811”美丽台州建设行动，深化环境综合整治提升、美丽公路建设和“两路两侧”“四边三化”工作，推进省级珍贵彩色森林示范市建设，加强近海海域环境整治，建设美丽海岸，深化长潭水库全国良好湖泊环境保护试点，积极创建生态文明建设示范区。

（四）聚焦深化改革，激发发展活力。

深化改革是台州赶超发展的不竭动力。要全面深化重要领域和关键环节改革，统筹“三去一降一补”，再创体制机制新优势。

不断深化国家级改革试点。继续深化国家级小微企业金融服务改革创新试验区建设，开展小微金融服务标准化试点，推动信保基金扩面，力争在保余额超50亿元、服务企业超5000家。积极引进外资银行和非银行类法人金融机构，加大股权基金引进力度，加快微金融小镇建设。推进农村合作金融机构股份制改革。实施商标品牌综合改革试点，深化商标专用权质押登记全国试点，开展股权、排污权、农村土地承包经营权等质押贷款。深化国家民间投资创新综合改革，用好用足PPP模式，使符合条件的重大项目采用PPP模式运作成为常态、使民资参与PPP项目成为常态，激发民间投资活力。深入推进国家循环经济示范城市、新型城镇化和信用体系建设示范城市等试点。

全面推进“最多跑一次”改革。深化“放管服”改革和“五张清单一张网”建设。坚持需求导向、问题导向、效果导向，按照“群众和企业到政府办事最多跑一次”的理念和目标，倒逼各级各部门减权、放权、治权，推进减事项、减材料、减环节、减时间、减收费，实行容缺受理。推行“一窗受理、集成服务”和“互联网+政务服务”，年底前实现“最多跑一次”覆盖80%以上行政事项，多让干部内部跑，多让数据网上跑，少让群众路上跑，确保政府办事效能明显提高，群众和企业获得感明显提升，区域发展环境明显改善，努力推动“最多跑一次”改革走在全省、全国前列。

统筹抓好关键领域改革。完善市区财政、土地管理体制，深化市区教育、卫生等体制改革，加快实现市区医疗一体化，推进市区社保和便民服务一体化。深化要素配置市场化改革，加快形成“亩产论英雄”机制。加快推进国资国企改革。实行耕地占补平衡全市域统筹。深化户籍制度改革，健全农村产权流转交易市场体系，推进农业转移人口市民化。统筹推进环保审批制度、网格化环境执法等省级改革试点。推进行政复议体制改革。推动公安机关大部门大警种制改革和各类事业单位统一登记管理试点。

（五）聚焦改善民生，增进人民福祉。

改善民生是台州赶超发展的出发点和落脚点。要坚持共享理念，将民生时刻放在心头、扛在肩上，让发展成果更多惠及群众。

加快发展社会事业。坚持优先发展教育，让更多孩子成就梦想、更多家庭实现希望。实行教育综合改革，全面推进教育现代化县（市、区）创建。大力发展幼儿教育，协调各类教育发展，提升教育质量。新建一批市区基础教育学校，推进北师大台州实验学校、国际学校建设。促进高等教育结构优化、内涵提升。积极引进优质高等教育资源，兴办与本地产业紧密衔接的高等院校。大力支持台州学院向应用型大学转型。加快发展职业教育，推动台州职业技术学院建设中德职业技术教育学院，加快台州科技职业学院二期和台州广播电视大学二期等建设，开工建设台州技师学院。推行“三医”联动，完成国家公立医院改革任务。深入推进“双下沉、两提升”，完善分级诊疗制度。完成市区三乙医院统一管理，争创三甲医院。大力发展中医药事业。建成启用市妇女儿童医院，加快建设市公共卫生中心、台州医院临海新院区，开工建设市护士学校，推动台州国际医院建设。提高出生人口素质，综合治理出生人口性别比。开展“2017台州文化年”系列文化活动，推进基层综合文化服务中心建设，提高基本公共文化服务标准化、均等化水平，推进国家公共文化服务体系示范区创建。加强和合文化研究推广，编写乡土教材，深入挖掘和合文化当代价值，弘扬和合文化精神。推动和合文化标志地、传播地建设，将其融入城市发展和地方产业品牌，建设天台山和合小镇、和合书院和一批主题公共设施。把和合文化融入旅游发展、文化传承、对外交流等方面，推动中华优秀传统文化走出去。加大文化产业发展力度。加强文化遗产传承保护利用和文艺精品、影视作品创作，支持台州乱弹发展。推动全民阅读，加强科学普及。建设台州大剧院、市民中心。推动市非遗展示馆、美术馆、民营经济博物馆和民办博物馆群建设。大力发展竞技体育、大众体育，创建体育强市。

积极促进就业和社会保障。就业是民生之本。实施更加积极的就业政策，推动大学生、农村转移劳动力、残疾人等重点人群创业就业，动态消除“零就业家庭”，新增城镇就业5.5万人。深入开展“台州无欠薪”行动，推进和谐劳动关系创建。加快各级各类社会保障制度整合，实现社会保障全覆盖、更公平、可持续。完善养老服务体系，鼓励引导社会力量参与养老机构运营。加快市社会福利院建设。推动精准扶贫、精准脱贫。实施集体经济薄弱村消除行动计划，多渠道增加农民收入，确保低收入农户人均可支配收入增长16%以上。进一步夯实统计基础，健全新兴产业统计。强化应急能力建设，提升突发事件应急处置能力。加强民族宗教、外事侨务工作。做好档案、地方志工作。继续做好对口支援帮扶工作。

坚决维护社会稳定。加强“平安台州”建设，实施“打霸拔钉、治乱除患、护航发展”平安台州2017系列行动，坚决打击阻挠、破坏经济建设的违法行为，严厉打击刑事犯罪。深化网格化管理，完善治安防控和公共安全体系，筑牢平安防线。人命关天，安全至上，要高度重视安全生产工作，铁腕开展“查危除患”大行动，深化消防、道路交通、渔业船舶、建筑施工等重点行业领域专项整治，坚决遏制重特大事故发生，确保安全生产控制指标持续下降。实施地质灾害综合治理三年行动计划。推进“七五”普法，加强法律援助和司法救助等公共法律服务体系建设。畅通信访渠道，完善社会矛盾源头预防和调处化解综合机制。加强基层政权建设，全面推进基层治理体系“四大平台”建设。食品药品安全事关人民健康，必须管得严而又严，要坚持源头控制、产管并重、重典治乱，全面启动国家食品安全城市创建。推动信用机制建设，加快建设覆盖全社会的诚信体系，打造信用台州。夯实基层反走私工作基础。严防涉渔“三无”船舶反弹，打赢“一打三整治”攻坚战。

继续办好十方面民生实事。一是加大环境治理力度。完成所有城镇污水处理设施一级A以上达标排放改造；新增污水管网200公里；污水处理率提高3个百分点。全面完成大型燃煤机组超低排放技术改造。全面淘汰改造燃煤小锅（窑）炉。加大畜禽养殖废弃物处理力度，完成大规模畜禽养殖场粪污处理设施建设。二是推进垃圾分类处理。研究制定推进垃圾分类的具体办法，加快城镇垃圾处理设施建设和垃圾填埋场生态化改造；推进农村垃圾“三化”处理扩面提升，基本实现农村生活垃圾分类收集集中处理全覆盖。三是提升食品安全水平。新建放心农贸市场35个；城区农贸市场快检体系建成率95%以上，追溯体系建成率95%以上。全市75%乡镇（街道）食安办完成规范化达标。小作坊登记率95%以上，产品监督抽检合格率92%以上，追溯率50%以上。四是加强交通治堵力度。深入实施城市交通拥堵治理，新增、更新公交车100辆，新建、改造公交站点100个，新增停车位10000个；实现全市域公交一卡通。开展市区“四小车”整治，推进城市道路人行过街设施建设。五是推进城乡惠民服务。新建城乡社区居家养老服务照料中心226个，实现农村社区居家养老服务照料中心全覆盖。提升美丽乡村公路600公里，新增绿道100公里。对457个村庄（含城中村）进行低压电网改造。对全市所有加油站加油机实施计量检定。六是丰富群众文体生活。新增农村文化礼堂150个、和合书吧（24小时自助图书馆）20个，创建“智慧”便民健身站点100个、便民足球场100个。七是促进教育事业发展。实施学前教育扩容工程，新建、改建二级以上标准幼儿园20所；新建、扩改建中小学生素质教育实践基地10个。八是加强公共安全管理。基本消除67个地质灾害隐患点；新建、扩改建避灾安置点111个；完成全部D级危房和涉及公共安全的C级危房治理；实施市区旧住宅区设施改造。全市所有社区完成消防安全社区创建。实施物联网电瓶车防盗项目。九是加快电商网络发展。新建260个农村电商服务站、320个城市社区智能投递终端。十是加强医疗卫生保障。推进全市 “健康一卡通”项目建设。实施职工医保居家医疗护理项目；巩固全科医生签约服务制度，城乡户籍重点人群签约率80%以上。完成8万名适龄妇女免费“两癌”筛查。完成11万名红十字公益性应急救护培训任务。

（六）聚焦自身建设，锻造台州铁军。

政府自身建设是实现赶超发展的坚强保证。要坚持党的领导，加快政府职能转变，提高行政效能，更好为人民服务。

建设学习型政府。认真学习党的路线方针政策，学习习近平总书记系列重要讲话精神和治国理政新理念新思想新战略，强化政治意识、大局意识、核心意识、看齐意识。加强法律法规和现代科学知识学习，完善知识结构，拓宽工作思路，提高科学履职能力。

建设服务型政府。按照“最多跑一次”要求进一步简政放权，能减则减、应放则放、该改则改，以政府权力的“减法”，换取群众满意度的“加法”，激发市场活力的“乘法”。切实加强公共服务、市场监管、社会管理、环境保护等职责，为基层和企业、群众排忧解难，当好企业和人民群众的“店小二”。

建设法治型政府。自觉接受市人大法律监督、市政协民主监督，认真办理人大议案建议和政协提案。坚持依法、民主、科学决策，完善重大行政决策制度。强化行政执法监督，提高依法执政水平。全面深化政务公开，主动接受社会监督、舆论监督。

建设廉洁型政府。坚持把纪律和规矩挺在前面，认真落实全面从严治党要求，深入推进廉政建设。严控“三公”经费支出，坚持政府过“紧日子”，让群众和企业过“好日子”。盯住行政审批、工程项目、惠民资金等重要领域和关键环节，加强行政监察和审计监督。持续改进作风，坚决治理“庸懒散奢”“中梗阻”现象，确保高效履职。

建设实干型政府。一分部署，九分落实。坚持求真务实、真抓实干，扑下身子抓落实。实施目标责任制管理，坚持领导率先垂范，压实工作责任，提高执行能力。加强督查考核、追责问责，给干事者鼓劲，为担当者撑腰。

各位代表！

奋斗托起使命，实干成就梦想。让我们紧密团结在以习近平同志为核心的党中央周围，在中共台州市委的坚强领导下，翻篇归零再出发，撸起袖子加油干，努力完成今年经济社会发展目标任务，加快建设独具魅力的“山海水城、和合圣地、制造之都”，以优异成绩迎接党的十九大胜利召开，为实现中华民族伟大复兴的中国梦而不懈奋斗！
